# Neuroinflammation predicts disease progression in progressive supranuclear palsy

**DOI:** 10.1136/jnnp-2020-325549

**Published:** 2021-03-17

**Authors:** Maura Malpetti, Luca Passamonti, Peter Simon Jones, Duncan Street, Timothy Rittman, Timothy D Fryer, Young T Hong, Patricia Vàsquez Rodriguez, William Richard Bevan-Jones, Franklin I Aigbirhio, John Tiernan O'Brien, James Benedict Rowe

**Affiliations:** 1 Department of Clinical Neurosciences and Cambridge University Hospitals NHS Trust, University of Cambridge, Cambridge, UK; 2 Institute of Molecular Bioimaging and Physiology, National Research Council, Milan, Italy; 3 Wolfson Brain Imaging Centre, University of Cambridge, Cambridge, UK; 4 Department of Psychiatry, University of Cambridge, Cambridge, UK; 5 MRC Cognition and Brain Sciences Unit, University of Cambridge, Cambridge, UK

## Abstract

**Introduction:**

In addition to tau pathology and neuronal loss, neuroinflammation occurs in progressive supranuclear palsy (PSP). However, the prognostic value of the in vivo imaging markers for these processes in PSP remains unclear. We test the primary hypothesis that baseline in vivo imaging assessment of neuroinflammation in subcortical regions predicts clinical progression in patients with PSP.

**Methods:**

Seventeen patients with PSP–Richardson’s syndrome underwent a baseline multimodal imaging assessment, including [^11^C]PK11195 positron emission tomography (PET) to index microglial activation, [^18^F]AV-1451 PET for tau pathology and structural MRI. Disease severity was measured at baseline and serially up to 4 years with the Progressive Supranuclear Palsy Rating Scale (PSPRS) (average interval of 5 months). Regional grey-matter volumes and PET ligand binding potentials were summarised by three principal component analyses (PCAs). A linear mixed-effects model was applied to the longitudinal PSPRS scores. Single-modality imaging predictors were regressed against the individuals’ estimated rate of progression to identify the prognostic value of baseline imaging markers.

**Results:**

PCA components reflecting neuroinflammation and tau burden in the brainstem and cerebellum correlated with the subsequent annual rate of change in the PSPRS. PCA-derived PET markers of neuroinflammation and tau pathology correlated with regional brain volume in the same regions. However, MRI volumes alone did not predict the rate of clinical progression.

**Conclusions:**

Molecular imaging with PET for microglial activation and tau pathology can predict clinical progression in PSP. These data encourage the evaluation of immunomodulatory approaches to disease-modifying therapies in PSP and the potential for PET to stratify patients in early phase clinical trials.

## Introduction

Neuroinflammation has been recognised as a common pathogenic process in progressive supranuclear palsy (PSP)^1 2^ and other tauopathies such as Alzheimer’s disease^3^ (AD) and frontotemporal dementia,^4–6^ together with genetic, epidemiological and imaging associations. For example, activated microglia are found in the neighbourhood of neurofibrillary tangles, even during early stages of disease,^1^ and are directly synaptotoxic.^3^ Neuroinflammation, including microglial activation, interacts with tau pathology to promote cell dysfunction and death in preclinical models of tauopathy.

Positron emission tomography (PET) radioligands have been developed to assess neuroinflammation and tau pathology accumulation in vivo in clinical cohorts. [^11^C]PK11195 is a widely used PET tracer that binds primarily to activated microglia in PSP^7–9^ and other neurodegenerative disorders.^10 11^ The ligand [^18^F]AV-1451 is widely used to assess tau pathology in AD and can be informative in PSP (see^12^ for review) despite lower sensitivity to PSP tau isoforms and off-target binding in some regions.^13 14^ However, it has not been shown whether either of these PET biomarkers of neuroinflammation and tau pathology predict longitudinal clinical progression in patients with PSP.

Our main hypothesis was that inflammation in the subcortical regions associated with PSP pathology promotes disease progression. We therefore test whether baseline in vivo measures of neuroinflammation ([^11^C]PK11195 PET) predict the annual rate of clinical progression in patients with PSP–Richardson’s syndrome. We test secondary hypotheses regarding the predictive value of baseline cortical inflammation, tau pathology ([^18^F]AV-1451 PET) and regional brain volume (structural MRI).

## Methods

### Participants

As part of the Neuroimaging of Inflammation in Memory and Other Disorders study,^15^ we recruited 17 people with a clinical diagnosis of probable PSP according to Movement Disorder Society (MDS) 1996 criteria.^16^ These participants also met the later MDS-PSP 2017 criteria for PSP–Richardson’s syndrome.^17^ Participants underwent a baseline neuropsychological assessment followed by an MRI scan and two PET scans with [^11^C]PK11195 (^11^C-labelled R-enantiomer of PK11195) and [^18^F]AV-1451 (also known as ^18^F-flortaucipir). The cross-sectional baseline data of [^11^C]PK11195 and [^18^F]AV-1451 have been published.^7^ Disease severity was measured at baseline visit and serially up to 4 years using the Progressive Supranuclear Palsy Rating Scale (PSPRS).^18^ Assessments were at an average of 5-month intervals (SD±2.3 months). Postmortem confirmation of PSP pathology was available in each of the eight patients who subsequently donated their brain to the Cambridge Brain Bank. For all 17 participants, the clinical diagnosis was reviewed and confirmed at follow-up.

### MRI and PET data acquisition and preprocessing

Full details of the imaging protocols have been published elsewhere.^9 19^ In brief, patients underwent 3 T MRI, together with dynamic PET imaging of [^11^C]PK11195 and [^18^F]AV-1451 for 75 and 90 min, respectively. MP-RAGE (Magnetization Prepared Rapid Acquisition Gradient Echo) T1-weighted MRI was acquired on Siemens Magnetom Tim Trio and Verio scanners (Siemens Healthineers, Erlangen, Germany), while PET scans were performed on a GE Advance and GE Discovery 690 PET/CT (GE Healthcare, Waukesha, USA). The two PET scanners used identical emission data acquisition protocols and image reconstruction algorithms (see Malpetti *et al* for discussion and more details^7^). Each patient underwent both [11C]PK11195 and [18F]AV-1451 PET scans using the same scanner (n=11 GE Discovery scanner, n=6 GE Advance scanner).^7^ Median (mean and SD) of the time interval between the baseline clinical assessment and the imaging scans were 0.0 (1.1±1.5) months for MRI, 2.0 (2.7±2.0) months for [^11^C]PK11195 PET and 1.0 (1.9±1.8) months for [^18^F]AV-1451 PET.

For each subject, the aligned dynamic PET image series for each scan was rigidly coregistered to the T1-weighted MRI image. Grey-matter volumes and non-displaceable binding potential (BP_ND_) values for each tracer were calculated in 83 cortical and subcortical regions of interest (ROIs) using a modified version of the Hammersmith atlas (www.brain-development.org), which includes parcellation of the brainstem and cerebellar dentate nucleus. Each T1 image was spatially normalised using Advanced Normalization Tools (http://www.picsl.upenn.edu/ANTS/), and the inverse transform was applied to a version of the Hammersmith atlas to bring ROIs to native T1 space. The T1-weighted images were segmented into grey matter, white matter and cerebrospinal fluid (CSF) with SPM12 (www.fil.ion.ucl.ac.uk) and were used to determine regional grey matter, white matter and CSF volumes, and to calculate the total intracranial volume (TIV=grey matter+white matter+CSF) in each participant. Regional grey-matter volumes included in further analyses were corrected for TIV. Prior to kinetic modelling, regional PET data were corrected for partial volume effects from the CSF by dividing by the mean regional grey-matter plus white-matter fraction determined from Statistical Parametric Mapping (SPM) segments smoothed to PET spatial resolution. For [^11^C]PK11195, supervised cluster analysis was used to determine the reference tissue time–activity curve, and BP_ND_ values were calculated in each ROI using a simplified reference tissue model with vascular binding correction.^20^ For [^18^F]AV-1451, BP_ND_ values were quantified in each ROI using a basis function implementation of the simplified reference tissue model,^21^ with superior cerebellar cortex grey matter as the reference region. This cerebellar region was selected as reference region given postmortem evidence showing minimal tau pathology in PSP (see pathology data in Passamonti *et al* 19).

### Statistical analyses

Grey-matter volumes and BP_ND_ values for each ligand were combined across the two hemispheres to derive 43 bilateral ROIs,^7 9 19^ which were next included in separate principal component analyses (PCAs) for each imaging modality. Varimax rotation was applied in each PCA to maximise interpretability and specificity of the resulting components. Components with eigenvalues of >1 were retained, explaining >80% of cumulative variance.

A linear mixed model was applied to longitudinal PSPRS scores collected from the first research visit to estimate the clinical annual rate of change at group level and then extract a patient-specific estimate of disease progression. The model included the estimation of a random intercept and slope, with time (in years) as independent variable and PSPRS scores as dependent variable. The effect of time on clinical changes has been also tested via likelihood ratio tests of the model described previously against the null model without the time effect. Linear mixed-effects analyses were performed using R software and lme4 package (R Core Team, 2012).

To test whether specific neuroanatomical patterns of grey-matter volume, microglial activation and tau pathology predict clinical progression, linear regression models were applied with the estimated rate of change (slope) as dependent variable, and each method-specific PCA component as predictor. First, we tested for significant regressions on slope with each modality-specific subcortical component as predictor, in accordance with our main hypothesis. Then, we explored the predictive value of cortical components running separate linear regression analyses for each imaging method and component. Age, education and sex were included as nuisance covariates. We tested associations between rate of change of clinical scores, duration from symptom onset to the baseline research visit, and the first PSPRS score.

Analogous linear regression models were then estimated with the intercept of the clinical severity as dependent variable. This identifies a cross-sectional association between imaging markers and clinical severity at baseline, which was estimated at individual level from the linear mixed-effects model on longitudinal PSPRS scores. For cross-sectional analyses, we expected to find significant associations with subcortical imaging components.^7 9 22 23^

Lastly, the modality-specific subcortical components were included in cross-modality Pearson correlations to test for associations between the strength of regional volumes, neuroinflammation and tau pathology.

### Data availability

Anonymised data may be shared by request to the senior author from a qualified investigator for non-commercial use (data sharing may be subject to restrictions according to consent and data protection legislation).

## Results

The demographics and clinical and cognitive variables of our sample are summarised in table 1. Fifteen out of 17 patients died within 5 years from the baseline research assessment (median=2.5 years, mean±SD=2.2 ± 1.0 (range 0.4–4.5) years from baseline visit). Eight of these 15 donated to the Cambridge Brain Bank, where post mortem examination confirmed PSP pathology in all eight. In table 1, we report demographic, clinical characteristics and group comparisons for two subgroups of patients, categorised by dividing the group along the median of time interval between study baseline and death. Age, years of education, baseline PSPRS and annual PSPRS rate of change were compared with independent-samples t-tests; sex was compared using χ^2^ test.

**Table 1:** Demographic and clinical characteristics

	Total patient group	Patient survival≤2.5 years	Patient survival>2.5 years	Difference
N	17	9	8	
Sex (F/M)	7/10	4/5	3/5	χ^2^=0.08, p=0.772
Age (mean±SD)	68.3±5.7	68.6±7.1	68.0±4.1	t(15)=0.20, p=0.848
Education (mean±SD)	12.1±1.9	11.8±1.8	12.5±2.1	t(15)=−0.77, p=0.452
Symptom duration (mean±SD)	4.7±1.5	4.6±1.7	4.8±1.5	t(15)=−0.28, p=0.786
PSPRS baseline (mean±SD)	41.2±14.5	45.1±14.8	37.0±13.7	t(15)=1.19, p=0.255
Clinical progression following baseline–PSPRS points/year (mean±SD)	6.2±1.5	6.4±0.7	5.9±2.1	t(15)=0.75, p=0.468

Statistics are reported for the total patient group and for subgroups based on median split of survival from baseline research assessment to death (median=2.5 years, mean±SD=2.2±1.0).

PSPRS, Progressive Supranuclear Palsy Rating Scale; t(), t-test.

### PCA of imaging data

**Figure 1:**
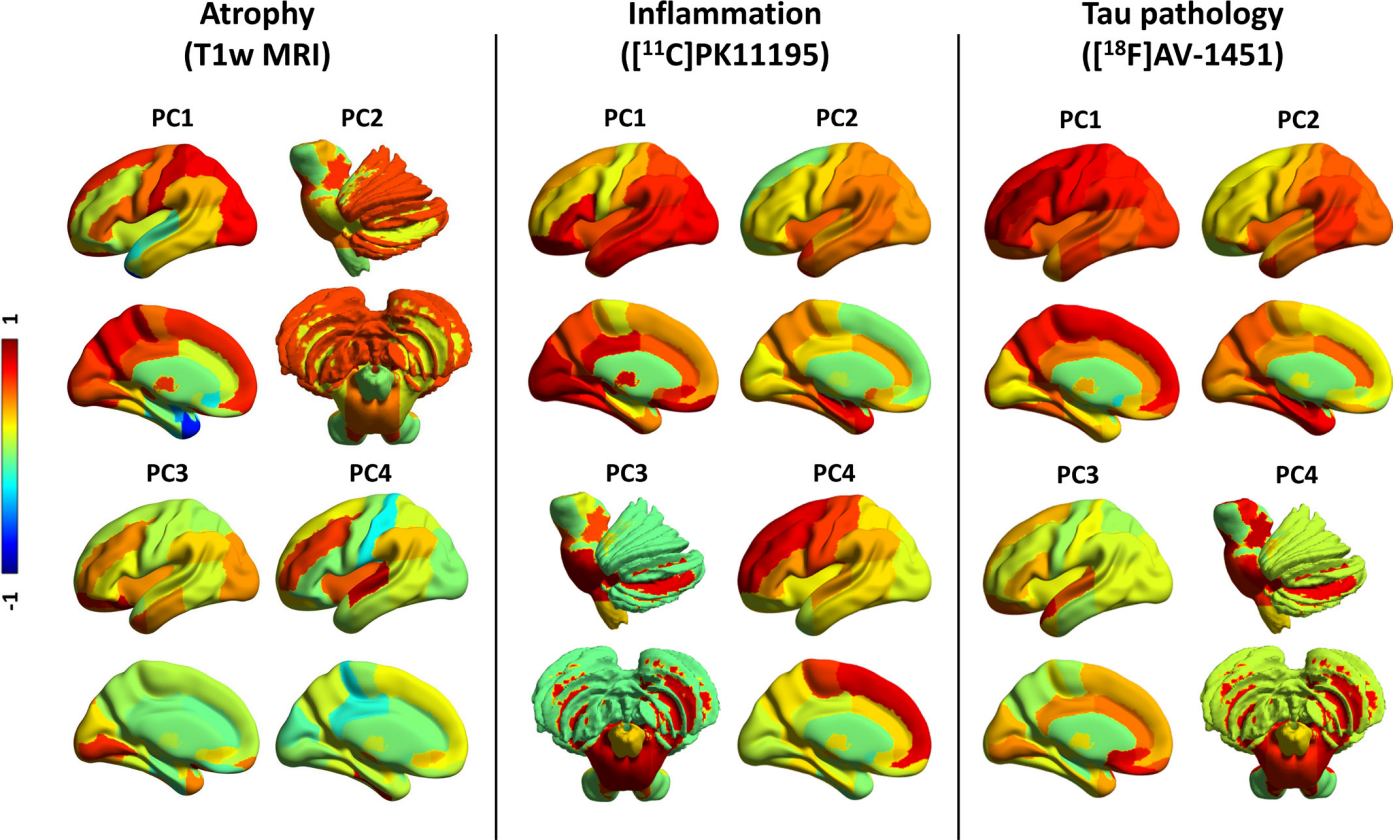
PCs for grey-matter volume, inflammation and tau pathology. First four PCs for grey-matter volumes (left panel), [^11^C]PK11195 BP_ND_ (middle panel) and [^18^F]AV-1451 BP_ND_ (right panel). The colours represent the rotated weights (range: from −1 to 1) of the brain regions for each component. BP_ND_, non-displaceable binding potential; PC, principal component.

For grey-matter volumes, seven components were identified, which explained 80.3% of the total variance. Figure 1 (left panel) provides a pictorial representation of the first four components, and online supplemental table 1 details regional weights in all seven components. Component 1 was widely distributed, including medial frontal cortex, thalamus, occipitoparietal regions, posterior cingulate cortex and postcentral cortex (32.0% of the total variance). Component 2 (11.8% variance) was weighted to midbrain, substantia nigra and pons, nucleus accumbens and putamen, as well as amygdala, hippocampus and precentral cortex, cerebellar grey-matter and dentate nucleus. Component 3 (10.0% variance) loaded onto the orbitofrontal cortex, anterior temporal lobe and lingual gyrus. Component 4 (7.9% variance) included the superior temporal gyrus, fusiform gyrus, middle inferior temporal lobe and insula.

10.1136/jnnp-2020-325549.supp1 Supplementary data

For [^11^C]PK11195 BP_ND_ and [^18^F]AV-1451 BP_ND_, each PCA identified four components, which collectively and respectively explained 81.4% and 81.8% of the data variance, as reported in Malpetti *et al*.^7^ For [^11^C]PK11195 (figure 1, middle panel; online supplemental table 2), component 1 loaded onto posterior cortical regions, the orbitofrontal cortex and cerebellar grey matter; component 2 grouped together medial and superior temporal lobes, insula and temporoparietal junction; component 3 was weighted to brainstem regions (ie, midbrain and pons), dentate nucleus and cerebellar white matter; while component 4 included superior and medial frontal regions. For [^18^F]AV-1451 (figure 1, right panel; online supplemental table 3), component 1 reflected global cortical binding; component 2 grouped insula and medial temporal lobe regions; component 3 loaded onto anterior superior temporal gyrus and frontal subgenual cortex; component 4 was weighted towards subcortical areas, including midbrain, pons, substantia nigra, thalamus, cerebellar dentate nucleus and white matter.

### Clinical progression as measured by longitudinal PSPRS

**Figure 2:**
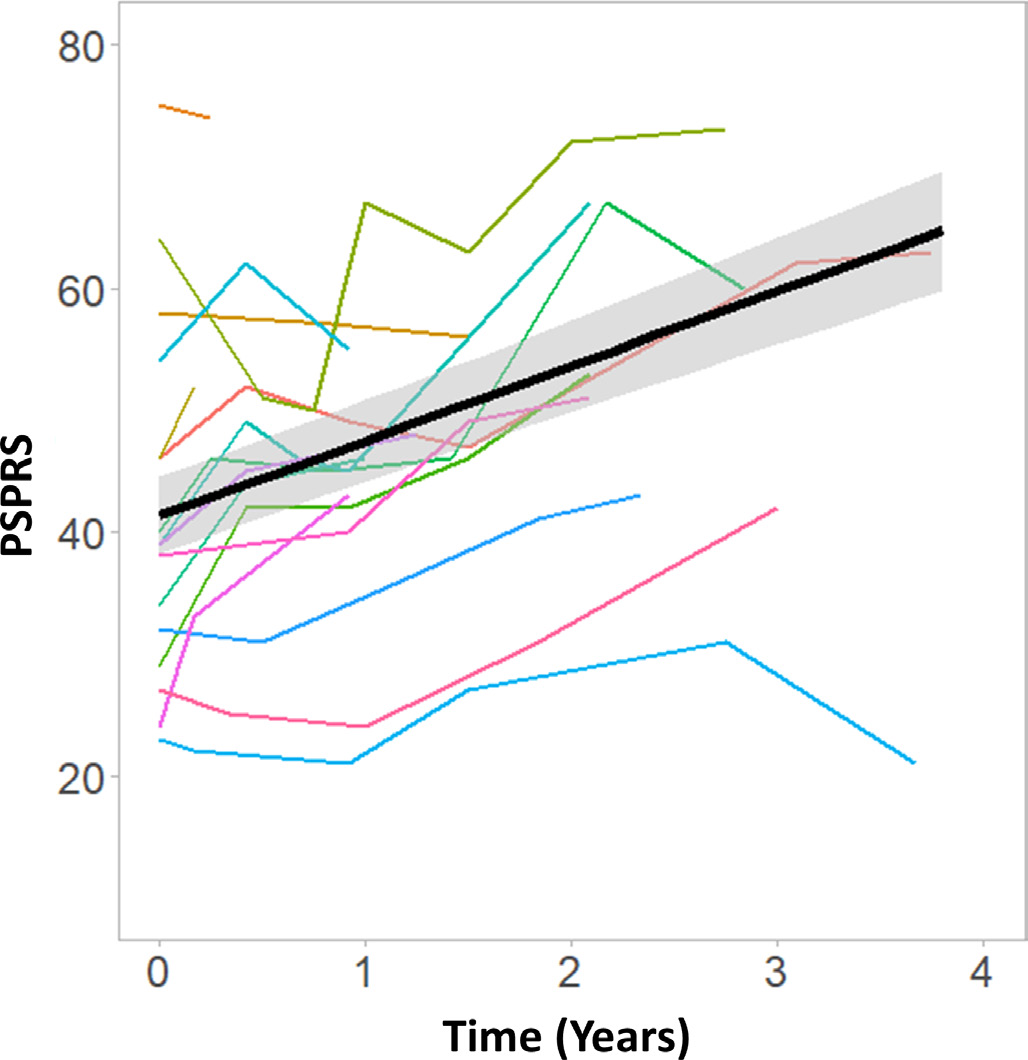
Clinical severity as measured by the PSPRS over time. Coloured lines chart the time course of the PSPRS score in individual patients. The black line represents the linear fit at group level. PSPRS, Progressive Supranuclear Palsy Rating Scale.

The linear mixed model on longitudinal PSPRS progression after baseline, considering the first research visit as baseline, indicated a significant effect of time (, ean=6.15 points/year, SD=1.06; figure 2 and table 1). The model comparison against the null model confirmed the significant effect of time (∆χ2=42.61, ∆df=3, p<0.0001). We also applied an analogous model over the whole longitudinal clinical assessment period, including all scores available from patients’ initial clinical diagnosis visit to their latest clinical visit. This confirmed a similar annual rate of change in PSPRS (mean=7.20 points/year, SD=1.18).

### Single-modality prediction models

**Figure 3:**
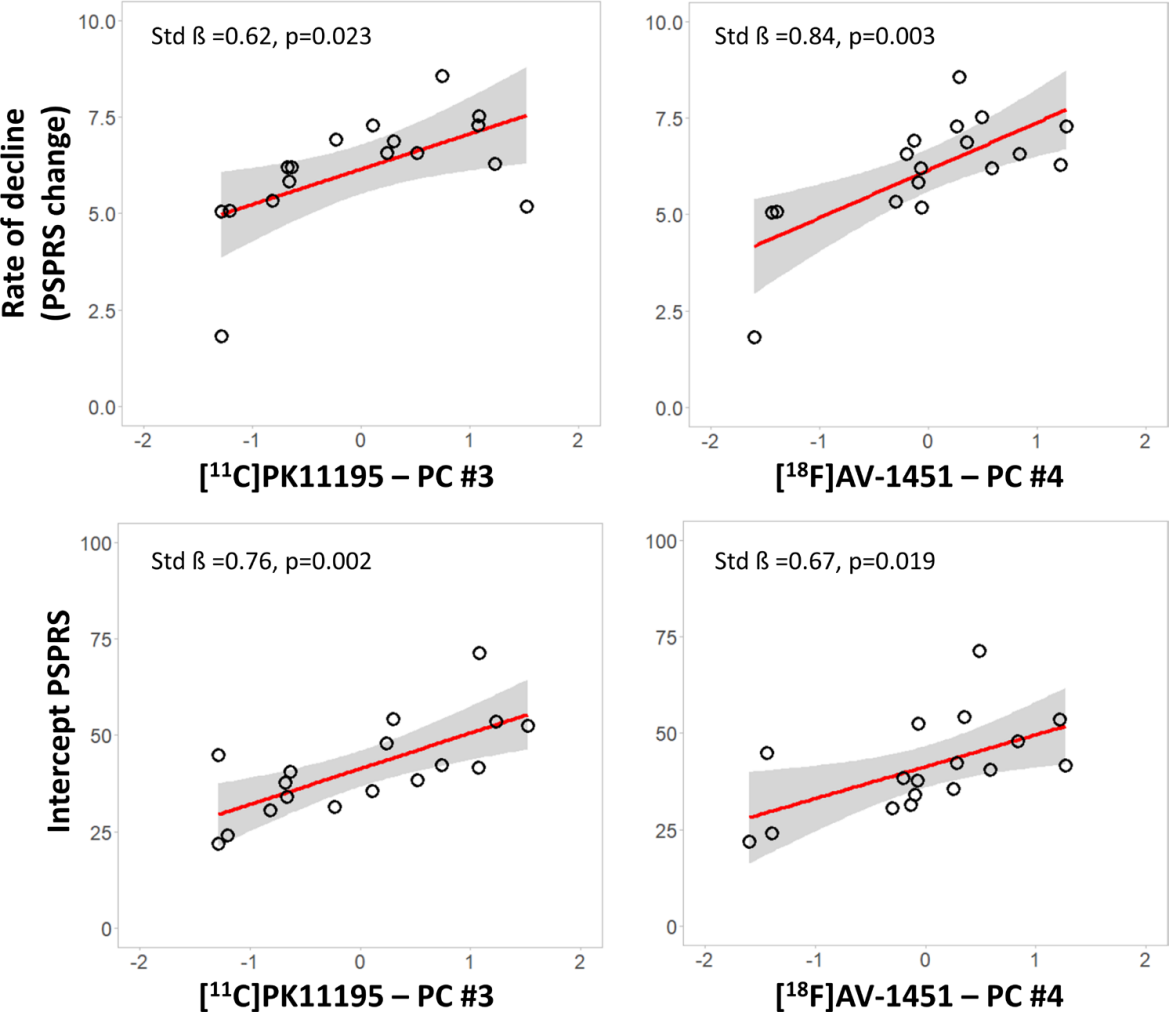
Inflammation and tau PET markers are associated with clinical severity and progression. Significant regression analyses of annual change in PSPRS scores (top row) and intercept PSPRS scores (bottom row) against baseline scores for each modality-specific principal component (X axis—residual component values corrected for covariates): [^11^C]PK11195 PET (left panel) and [^18^F]AV-1451 PET (right panel). Estimated parameters are reported for each model with age, education and sex as covariates. PET, positron emission tomography; PSPRS, Progressive Supranuclear Palsy Rating Scale.

We tested whether imaging markers in subcortical components predicted longitudinal PSPRS progression, applying linear regression models for each modality-specific subcortical components (MRI component 2, [^11^C]PK11195 component 3 and [^18^F]AV-1451 component 4). Correcting for age, education and sex, the annual rate of clinical progression was related positively with (1) the [^11^C]PK11195 subcortical component 3 (standardised beta=0.624, p=0.023) and (2) the [^18^F]AV-1451 subcortical component 4 (standardised beta=0.840, p=0.003) (figure 3, top row). Applying regression models on the slope with single cortical components, age, education and sex as predictors, we found that no other components had significant correlations with the annual rate of clinical progression (p>0.05 after false discovery rate (FDR) correction for multiple comparisons). For MRI, no component showed an association with clinical rate of change (p>0.05 after FDR correction for multiple comparisons). The regression model on clinical progression with symptom duration as single regressor was not significant (standardised beta=−0.06, p=0.822 uncorrected). Similarly, clinical severity at baseline (PSPRS) was not associated with clinical progression (standardised beta=0.33, p=0.196).

We tested whether imaging markers in subcortical components were related to baseline variation in disease severity. Across the three modality-specific subcortical components, linear regression models with individual PSPRS *intercept* scores as the dependent variable, correcting for age, education and sex, indicated significant associations with the [^11^C]PK11195 subcortical component #3 (standardised beta=0.755, p=0.002), and [^18^F]AV-1451 subcortical component #4 (standardised beta=0.673, p=0.019) (figure 3, bottom row). However, there was no significant association between intercept and any of the grey-matter components or cortical PET components (all p>0.05 after FDR correction for multiple comparisons). The regression model on clinical intercept scores with symptom duration (standardised beta=0.43, p=0.086) was not significant.

### Intermodality correlations between subcortical imaging components

Simple correlations across subjects between MRI and PET subcortical components of clinical slope were significant for MRI component #2 with [^11^C]PK11195 component 3 (r=−0.584, p=0.014 uncorrected, p=0.014 FDR correction) and [^18^F]AV-1451 component 4 (r=−0.626, p=0.007 uncorrected, p=0.011 FDR correction). The correlation between [^11^C]PK11195 component 3 and [^18^F]AV-1451 component 4 was also significant (r=0.769, p<0.0001 uncorrected, p<0.0001 FDR correction).^7^

## Discussion

Our main finding is that subcortical neuroinflammation is associated with clinical severity of PSP at baseline, and with faster subsequent clinical progression. A similar effect is found for estimated subcortical tau pathology, with the caveats related to interpreting [^18^F]AV1451 binding in PSP. The PET markers were associated with each other and with structural MRI measures for atrophy in the same regions. However, subcortical grey-matter volumes were not correlated with subsequent clinical progression and were not significantly related to clinical severity at baseline. Similarly, clinical severity at baseline was not predictive of clinical progression in the following years, suggesting that the annual rate of clinical changes is approximately constant throughout different stages of disease.

Several studies in PSP have explored the association between changes in clinical severity and in vivo neuroimaging markers for microglial activation (eg, [^11^C]PK11195 PET^8 9^), tau pathology (eg, [^18^F]AV-1451 PET^19 22–25^) and atrophy (eg, structural MRI^26–28^). This study, however, focusses on the prognostic (or predictive) potential of baseline multimodal imaging markers. The baseline uptakes of both [^11^C]PK11195 and [^18^F]AV-1451 in PSP-related subcortical regions correlated with the subsequent annual rate of change in severity, as measured by PSPRS. Note that we are not testing whether the progression of PET markers compares with progression of disease severity. The progression of [^18^F]AV-1451 uptake has been compared with MRI-derived atrophy progression,^29^ revealing greater atrophy changes than PET signal changes over time. Instead, for prognostic value, we found that grey-matter volumetric measures were weaker predictors than PET markers, despite the correlation of subcortical grey-matter volumes with [^11^C]PK11195 and [^18^F]AV-1451 binding. The latter correlation suggests a close relationship between not only microglial activation and tau pathology,^1 7^ but also with neurodegeneration in those subcortical regions commonly associated with pathological hallmarks of PSP.^30^ Our findings align with studies of AD, another tauopathy, in which baseline in vivo PET markers of tau pathology and microglial activation predicted clinical progression, outperforming structural MRI.^31^

The correlation of in vivo PET measures with baseline disease severity has been reported in previous studies.^7 9 22 23^ Using [^11^C]PK11195 PET to assess neuroinflammation, a study reported a positive association was observed between clinical severity and ligand binding in pallidum, midbrain and pons.^9^ There was a strong association between [^11^C]PK11195 binding and [^18^F]AV-1451 in these subcortical regions, although inconsistent findings are reported in studies using [^18^F]AV-1451in PSP (see Leuzy *et al* 12). The sporadic lack of significant correlates of [^18^F]AV-1451 in PSP is often attributed to the low affinity of the ligand for 4R-tau pathology. However, in relatively small studies, sessional variance of clinical rating scales may also reduce power. Therefore, our estimate of baseline clinical severity used the intercept extracted from the linear mixed-effects model of longitudinal PSPRS scores rather than single baseline assessment.

The null result for structural MRI predictors might be surprising, given previous reports on the utility of visual and volumetric atrophy assessments in the midbrain and other subcortical regions, including caudate, putamen, globus pallidus, subthalamus and thalamus, as an in vivo biomarker in patients with PSP.^32^ Indeed, structural MRI has provided the most studied and validated diagnostic biomarkers in PSP. However, a biomarker’s properties for diagnostics (ie, presence of PSP^32 33^) or correlates of severity (ie, at baseline) do not imply the property of prognostication.

Overall, our findings on the in vivo association between imaging markers of different pathological processes and their prognostic relevance accord with postmortem data^1 2^ and suggest a key role for microglial activation and tau burden on neurodegeneration, and consequent clinical progression. A growing literature supports a role for neuroinflammation in driving tau spreading and neurodegeneration in tauopathies (see Vogels *et al* 3 for review). Furthermore, genome-wide association studies implicate inflammatory pathways in the aetiology of tauopathies.^34 35^ For example, Jabbari *et al* reported an association between a common variation at the leucine-rich repeat kinase 2 (LRRK2) locus and survival from symptom onset to death in patients with PSP.^35^ This relationship may be mediated by the effect of increased LRRK2 expression in microglia proinflammatory responses,^36^ promoting spread and accumulation of misfolded tau protein, analogous to AD.^3^ This hypothesis is supported by the association of dysregulated expression of the microglial-related gene CXCR4, regional accumulation of neurofibrillary tangles and increased risk of PSP.^37^ The role of early neuroinflammation in tauopathies is supported by PET evidence of microglial activation preceding PET evidence of aggregated tau and symptoms in carriers of mutations of the microtubule associated protein tau gene (MAPT).^5^ A preliminary study of longitudinal changes in microglial activation in two patients with PSP showed stable microglial activation across 6–10 months^8^ but may have lacked power. However, preclinical evidence with PET in tau transgenic mice suggests that inflammation increases longitudinally and predicts greater tau accumulation and lesser performance over time.^38^

There are several limitations to our study. We recruited according to clinical diagnostic criteria, and although clinicopathological correlations of PSP-Richardson’s syndrome are very high, including 8 of 8 cases in our study with post mortem pathology, they are not perfect. Moreover, the average rate of change in severity was 6–7 points per year on the PSPRS, which is lower than several previous observational studies^18 39^ and clinical trials.^40 41^ This may partially be due to selection criteria that favoured patients robust enough to undergo three brain scans at baseline. However, our cases were otherwise typical, and 15 out of 17 died within 5 years from baseline (mean 2.2 years±1.0). The modest size of our cohort prevented the application of complex models for the direct comparison between MRI and PET predictors, such as multiple linear regression or linear mixed models with several independent variables. The replication of these findings with larger and multicenter clinical cohorts will be important to establish generalisability of our results. Other limitations relate to the PET tracers used. [^11^C]PK11195 binds to the 18 kDa translocator protein which is overexpressed in activated microglia, but also in other cell types, like astrocytes and vascular smooth muscle cells, although it has been found selective for activated microglia over reactive astrocytes.^42^ There are also caveats for [^18^F]AV-1451, namely, its off-target binding (monoamine oxidase, choroid plexus and neuromelanin) and lower affinity for PSP tau compared with AD-related tau (see Malpetti *et al* and Leuzy *et al* 7 12 for discussion regarding limitations of this ligand). Nonetheless, the topological distribution of [^18^F]AV-1451 binding and correlations with severity maintain utility for this ligand even in PSP.

In conclusion, our results support the relevance of neuroinflammation for progression of PSP–Richardson’s syndrome. We suggest that [^11^C]PK11195 may be a valuable biomarker for clinical trials in PSP, complementary to structural MRI. The PET markers may be useful for stratification of patients based on prognosis and for evaluation of therapeutic response, supporting the development of immunomodulatory strategies for disease-modifying treatments in PSP, alone or in conjunction with treatments directed against tau and other pathogenic pathways.

## Data Availability

Data are available upon reasonable request. Anonymised data may be shared by request to the senior author from a qualified investigator for non-commercial use (data sharing may be subject to restrictions according to consent and data protection legislation).
