# Supplementary material for: Neuroinflammation predicts disease progression in progressive supranuclear palsy
Source: J Neurol Neurosurg Psychiatry. 2021 Mar 17;92(7):769–75. doi: 10.1136/jnnp-2020-325549 (PMC7611006; doi:10.1136/jnnp-2020-325549)
Supplement: Supplementary data [file jnnp-2020-325549supp001.pdf]

# Supplementary Material

**eTable 1.** Regional rotated weights of the seven significant MRI principal components (PC) identified by principal component analysis on grey-matter regional volumes.

| ***Regions*** | **PC1** | **PC2** | **PC3** | **PC4** | **PC5** | **PC6** | **PC7** |
|---|---|---|---|---|---|---|---|
| *Postcentral_gyrus* | 0.871 | 0.101 | 0.115 | -0.227 | 0.151 | 0.132 | -0.003 |
| *Superior_parietal_gyrus* | 0.769 | 0.082 | 0.077 | 0.167 | 0.037 | -0.038 | 0.290 |
| *Lateral_remainder_of_occipital_lobe* | 0.742 | -0.085 | 0.449 | 0.020 | 0.111 | 0.140 | 0.135 |
| *Anterior_temporal_lobe_medial_part* | -0.710 | 0.118 | 0.488 | 0.128 | 0.177 | -0.127 | -0.100 |
| *Medial_orbital_gyrus* | 0.693 | 0.208 | 0.499 | 0.148 | 0.294 | -0.175 | 0.202 |
| *Superior_frontal_gyrus* | 0.672 | 0.039 | 0.114 | 0.234 | 0.616 | 0.226 | 0.018 |
| *Thalamus* | 0.646 | 0.391 | 0.116 | 0.165 | -0.309 | 0.140 | -0.377 |
| *Cuneus* | 0.633 | -0.037 | 0.294 | -0.072 | 0.268 | -0.296 | -0.054 |
| *Gyrus_cinguli_posterior_part* | 0.598 | 0.254 | 0.004 | -0.164 | -0.076 | -0.126 | 0.358 |
| *Caudate_nucleus* | 0.588 | 0.293 | 0.256 | -0.100 | 0.214 | 0.383 | 0.004 |
| *Inferior_frontal_gyrus* | 0.546 | 0.434 | 0.225 | 0.017 | 0.041 | 0.254 | 0.536 |
| *Cerebellum_dentate* | 0.205 | 0.894 | -0.037 | -0.071 | 0.080 | -0.054 | -0.020 |
| *Nucleus_accumbens* | 0.029 | 0.814 | 0.075 | 0.016 | 0.181 | 0.032 | 0.000 |
| *Amygdala* | -0.421 | 0.790 | 0.262 | 0.261 | 0.051 | -0.037 | -0.029 |
| *Brainstem_mid* | 0.104 | 0.656 | 0.082 | 0.491 | -0.052 | 0.203 | 0.099 |
| *Substantia_nigra* | 0.311 | 0.653 | 0.003 | 0.505 | -0.151 | -0.034 | 0.114 |
| *Putamen* | 0.513 | 0.638 | 0.062 | 0.053 | -0.078 | 0.382 | 0.043 |
| *Hippocampus* | -0.002 | 0.610 | -0.004 | 0.149 | 0.503 | 0.482 | -0.088 |
| *Precentral_gyrus* | 0.526 | 0.608 | 0.110 | 0.051 | 0.238 | -0.162 | 0.223 |
| *Cerebellum_gm* | 0.319 | 0.607 | 0.288 | 0.071 | 0.249 | 0.217 | -0.015 |
| *Posterior_orbital_gyrus* | 0.075 | 0.152 | 0.872 | 0.033 | -0.052 | 0.100 | 0.324 |
| *Anterior_orbital_gyrus* | 0.289 | 0.300 | 0.710 | 0.305 | 0.392 | 0.144 | -0.037 |
| *Lingual_gyrus* | 0.574 | -0.048 | 0.660 | 0.048 | 0.020 | 0.176 | 0.022 |
| *Anterior_temporal_lobe_lateral_part* | 0.124 | 0.063 | 0.605 | 0.062 | -0.029 | 0.233 | -0.063 |
| *Brainstem_pon* | -0.255 | 0.505 | -0.573 | -0.204 | 0.045 | 0.163 | 0.329 |
| *Superior_temporal_gyrus_posterior_part* | -0.084 | 0.272 | 0.207 | 0.802 | 0.221 | 0.107 | -0.027 |
| *Fusiform_gyrus* | -0.279 | -0.055 | -0.136 | 0.783 | -0.056 | -0.225 | 0.088 |
| *Middle_and_inferior_temporal_gyrus* | 0.155 | 0.112 | 0.460 | 0.628 | 0.253 | 0.139 | -0.027 |
| *Insula* | 0.174 | 0.120 | 0.450 | 0.558 | 0.046 | -0.075 | 0.269 |
| *Parahippocampal_and_ambient_gyri* | 0.065 | 0.099 | -0.008 | 0.098 | 0.960 | -0.050 | -0.052 |
| *Subcallosal_area* | 0.132 | 0.204 | 0.209 | -0.026 | 0.761 | 0.079 | 0.070 |
| *Brainstem_med* | 0.140 | 0.034 | -0.102 | -0.037 | -0.013 | 0.939 | 0.107 |
| *Lateral_orbital_gyrus* | 0.057 | 0.118 | 0.334 | -0.024 | 0.055 | 0.757 | -0.049 |
| *Presubgenual_frontal_cortex* | 0.124 | -0.041 | 0.256 | 0.368 | 0.125 | 0.545 | 0.470 |
| *Superior_temporal_gyrus_anterior_part* | -0.201 | -0.002 | 0.062 | 0.097 | 0.061 | 0.005 | -0.839 |
| *Subgenual_frontal_cortex* | -0.231 | -0.051 | 0.301 | 0.316 | 0.072 | 0.301 | 0.671 |
| *Inferiolateral_remainder_of_parietal_lobe* | 0.401 | 0.042 | 0.323 | 0.350 | -0.149 | -0.171 | 0.616 |
| *Cerebellum_wm* | 0.078 | 0.197 | 0.072 | -0.148 | 0.234 | 0.061 | -0.142 |
| *Cingulate_gyrus_anterior_part* | 0.164 | 0.291 | -0.031 | 0.004 | -0.279 | 0.124 | 0.250 |
| *Straight_gyrus* | 0.085 | -0.062 | -0.065 | 0.080 | 0.101 | -0.039 | 0.085 |
| *Middle_frontal_gyrus* | 0.319 | 0.338 | 0.427 | 0.122 | 0.176 | -0.278 | 0.142 |
| *Pallidum* | -0.030 | 0.374 | -0.149 | -0.034 | 0.216 | 0.031 | 0.170 |
| *Posterior_temporal_lobe* | 0.324 | 0.151 | 0.166 | 0.175 | 0.077 | 0.121 | 0.397 |
| ***Cumulative % of Variance*** | **32.0** | **43.8** | **53.8** | **61.6** | **68.8** | **75.2** | **80.8** |

**eTable 2.** Regional rotated weights of the four significant [$^{11}$C]PK11195 PET principal components (PC) identified by principal component analysis on non-displaceable binding potential regional values.

| ***Regions*** | **PC 1** | **PC 2** | **PC 3** | **PC 4** |
|---:|---|---|---|---|
| *Lingual_gyrus* | 0.903 | 0.297 | 0.145 | 0.127 |
| *Cerebellum_gm* | 0.900 | 0.173 | -0.034 | 0.156 |
| *Anterior_orbital_gyrus* | 0.897 | 0.104 | 0.288 | 0.184 |
| *Cuneus* | 0.892 | 0.232 | 0.132 | 0.157 |
| *Medial_orbital_gyrus* | 0.869 | 0.233 | 0.196 | 0.269 |
| *Straight_gyrus* | 0.861 | 0.370 | 0.066 | 0.169 |
| *Lateral_orbital_gyrus* | 0.827 | 0.351 | 0.019 | 0.295 |
| *Thalamus* | 0.820 | 0.124 | 0.203 | 0.294 |
| *Gyrus_cinguli_posterior_part* | 0.816 | 0.299 | 0.017 | 0.165 |
| *Caudate_nucleus* | 0.814 | 0.086 | 0.069 | -0.002 |
| *Presubgenual_frontal_cortex* | 0.786 | 0.161 | 0.211 | 0.355 |
| *Inferior_frontal_gyrus* | 0.779 | 0.273 | 0.059 | 0.447 |
| *Middle_and_inferior_temporal_gyrus* | 0.774 | 0.495 | 0.206 | 0.277 |
| *Lateral_remainder_of_occipital_lobe* | 0.762 | 0.437 | 0.287 | 0.190 |
| *Fusiform_gyrus* | 0.758 | 0.371 | 0.260 | -0.076 |
| *Anterior_temporal_lobe_lateral_part* | 0.745 | 0.547 | 0.147 | 0.126 |
| *Posterior_orbital_gyrus* | 0.740 | 0.490 | 0.223 | 0.205 |
| *Posterior_temporal_lobe* | 0.723 | 0.522 | 0.287 | 0.304 |
| *Subgenual_frontal_cortex* | 0.691 | 0.363 | 0.350 | 0.279 |
| *Superior_temporal_gyrus_posterior_part* | 0.687 | 0.398 | 0.187 | 0.423 |
| *Inferiolateral_remainder_of_parietal_lobe* | 0.674 | 0.499 | 0.117 | 0.391 |
| *Superior_parietal_gyrus* | 0.610 | 0.464 | 0.112 | 0.291 |
| *Insula* | 0.586 | 0.560 | 0.362 | 0.253 |
| *Cingulate_gyrus_anterior_part* | 0.579 | 0.413 | -0.127 | 0.318 |
| *Subcallosal_area* | 0.567 | 0.555 | -0.025 | -0.086 |
| *Anterior_temporal_lobe_medial_part* | 0.475 | 0.798 | 0.051 | 0.025 |
| *Hippocampus* | 0.235 | 0.794 | 0.311 | 0.170 |
| *Amygdala* | 0.238 | 0.789 | 0.351 | 0.202 |
| *Superior_temporal_gyrus_anterior_part* | 0.645 | 0.670 | -0.011 | 0.145 |
| *Parahippocampal_and_ambient_gyri* | 0.513 | 0.668 | 0.225 | 0.279 |
| *Nucleus_accumbens* | 0.443 | 0.567 | -0.237 | 0.166 |
| *Brainstem_pon* | 0.304 | 0.129 | 0.876 | 0.061 |
| *Cerebellum_dentate* | 0.067 | -0.030 | 0.847 | 0.198 |
| *Cerebellum_wm* | 0.089 | 0.365 | 0.797 | -0.015 |
| *Brainstem_mid* | 0.436 | 0.233 | 0.608 | 0.390 |
| *Superior_frontal_gyrus* | 0.426 | 0.012 | -0.004 | 0.792 |
| *Middle_frontal_gyrus* | 0.298 | 0.285 | 0.359 | 0.778 |
| *Precentral_gyrus* | 0.205 | 0.315 | 0.451 | 0.634 |
| *Postcentral_gyrus* | 0.402 | 0.455 | 0.115 | 0.622 |
| *Substantia_nigra* | -0.051 | 0.113 | 0.074 | 0.026 |
| *Brainstem_med* | 0.386 | 0.234 | 0.331 | -0.026 |
| *Putamen* | 0.620 | 0.099 | 0.008 | 0.043 |
| *Pallidum* | -0.030 | 0.138 | 0.473 | 0.120 |
| ***Cumulative % of Variance*** | **62.8** | **72.0** | **77.1** | **81.4** |

**eTable 3.** Regional rotated weights of the four significant [$^{18}$F]AV-1451 PET principal components (PC) identified by principal component analysis on non-displaceable binding potential regional values.

| ***Regions*** | **PC 1** | **PC 2** | **PC 3** | **PC 4** |
|---|---|---|---|---|
| *Middle_frontal_gyrus* | 0.891 | 0.278 | 0.177 | 0.210 |
| *Postcentral_gyrus* | 0.791 | 0.465 | 0.231 | 0.159 |
| *Precentral_gyrus* | 0.769 | 0.355 | 0.073 | 0.392 |
| *Superior_frontal_gyrus* | 0.766 | 0.241 | 0.382 | 0.225 |
| *Lateral_orbital_gyrus* | 0.731 | 0.437 | 0.200 | 0.174 |
| *Inferior_frontal_gyrus* | 0.711 | 0.305 | 0.384 | 0.410 |
| *Inferiolateral_remainder_of_parietal_lobe* | 0.706 | 0.620 | 0.173 | 0.202 |
| *Superior_parietal_gyrus* | 0.704 | 0.567 | 0.077 | 0.029 |
| *Middlend_inferior_temporal_gyrus* | 0.656 | 0.511 | 0.078 | 0.388 |
| *Lateral_remainder_of_occipital_lobe* | 0.645 | 0.550 | 0.193 | 0.330 |
| *Medial_orbital_gyrus* | 0.584 | 0.107 | 0.572 | 0.415 |
| *Superior_temporal_gyrus_posterior_part* | 0.577 | 0.380 | 0.545 | 0.301 |
| *Anterior_orbital_gyrus* | 0.533 | 0.106 | 0.517 | 0.497 |
| *Amygdala* | 0.240 | 0.878 | 0.283 | 0.131 |
| *Hippocampus* | 0.281 | 0.825 | -0.016 | 0.341 |
| *Parahippocampal_and_ambient_gyri* | 0.410 | 0.767 | 0.326 | -0.038 |
| *Anterior_temporal_lobe_medial_part* | 0.307 | 0.758 | 0.422 | -0.045 |
| *Fusiform_gyrus* | 0.355 | 0.752 | 0.178 | 0.064 |
| *Brainstem_med* | 0.086 | 0.736 | 0.133 | 0.299 |
| *Anterior_temporal_lobe_lateral_part* | 0.449 | 0.682 | 0.301 | 0.188 |
| *Posterior_temporal_lobe* | 0.559 | 0.668 | 0.200 | 0.358 |
| *Posterior_orbital_gyrus* | 0.565 | 0.636 | 0.344 | 0.269 |
| *Insula* | 0.540 | 0.554 | 0.225 | 0.457 |
| *Cingulate_gyrus_anterior_part* | 0.464 | 0.551 | 0.511 | -0.074 |
| *Subcallosal_area* | -0.206 | 0.189 | 0.840 | -0.029 |
| *Superior_temporal_gyrus_anterior_part* | 0.396 | 0.334 | 0.794 | 0.164 |
| *Subgenual_frontal_cortex* | 0.313 | 0.236 | 0.785 | 0.289 |
| *Straight_gyrus* | 0.344 | 0.400 | 0.732 | 0.081 |
| *Presubgenual_frontal_cortex* | 0.418 | 0.086 | 0.700 | 0.318 |
| *Cerebellum_dentate* | 0.003 | -0.007 | 0.193 | 0.907 |
| *Brainstem_mid* | 0.221 | 0.227 | 0.136 | 0.872 |
| *Cerebellum_wm* | 0.524 | 0.121 | -0.081 | 0.770 |
| *Thalamus* | 0.395 | 0.229 | 0.285 | 0.726 |
| *Brainstem_pon* | 0.262 | 0.519 | 0.193 | 0.679 |
| *Substantia_nigra* | 0.457 | 0.274 | 0.099 | 0.547 |
| *Putamen* | 0.223 | 0.296 | -0.011 | 0.296 |
| *Caudate_nucleus* | 0.084 | 0.061 | 0.553 | 0.301 |
| *Nucleusccumbens* | 0.389 | 0.402 | 0.255 | 0.066 |
| *Lingual_gyrus* | 0.246 | 0.419 | 0.477 | 0.210 |
| *Pallidum* | 0.274 | 0.133 | 0.227 | 0.473 |
| *Cuneus* | 0.321 | 0.482 | 0.376 | 0.279 |
| *Gyrus_cinguli_posterior_part* | 0.504 | 0.450 | 0.453 | 0.116 |
| *Cerebellum_gm* | -0.038 | 0.032 | 0.009 | 0.135 |
| ***Cumulative % of Variance*** | **61.3** | **69.9** | **76.9** | **81.8** |
